# Persistence and adverse events of biological treatment in adult patients with juvenile idiopathic arthritis: results from BIOBADASER

**DOI:** 10.1186/s13075-018-1728-3

**Published:** 2018-10-10

**Authors:** Juan José Bethencourt Baute, Carlos Sanchez-Piedra, Dolores Ruiz-Montesinos, Marta Medrano San Ildefonso, Carlos Rodriguez-Lozano, Eva Perez-Pampin, Ana Ortiz, Sara Manrique, Rosa Roselló, Victoria Hernandez, Cristina Campos, Agustí Sellas, Walter Alberto Sifuentes-Giraldo, Javier García-González, Fernando Sanchez-Alonso, Federico Díaz-González, Juan Jesús Gómez-Reino, Sagrario Bustabad Reyes, on behalf of the BIOBADASER study group

**Affiliations:** 1 Servicio de Reumatología, Hospital Universitario de Canarias, Tenerife, Spain; 2 Research Unit, Sociedad Española de Reumatología, Madrid, Spain; 3 Servicio de Reumatología, Hospital Universitario del Virgen Macarena, Sevilla, Spain; 4 Servicio de Reumatología Hospital Universitario Miguel Servet, Zaragoza, Spain; 5 Servicio de Reumatología, Hospital Universitario de Gran Canaria Dr. Negrín, Las Palmas, Spain; 6 Servicio de Reumatología, Hospital Clínico Universitario de Santiago, A Coruña, Spain; 7 Servicio de Reumatología, Hospital de La Princesa, Madrid, Spain; 8 UGC de Reumatología, Instituto de Investigación Biomédica de Málaga (IBIMA), Hospital Regional Universitario de Málaga, Universidad de Málaga, Málaga, Spain; 9 Servicio de Reumatología, Hospital San Jorge, Huesca, Spain; 10 Servicio de Reumatología, Hospital Clinic de Barcelona, Barcelona, Spain; 11 Servicio de Reumatología, Hospital General Universitario Valencia, Valencia, Spain; 12 Servicio de Reumatología, Hospital Vall d’Hebron, Barcelona, Spain; 13 Servicio Reumatología, Hospital Ramón y Cajal, Madrid, Spain; 14 Servicio de Reumatología, Hospital 12 de Octubre, Madrid, Spain; 15 Servicio de Reumatología, Hospital Universitario de Canarias, c/Ofra s/n 38320, La Laguna, Santa Cruz de Tenerife, Spain

**Keywords:** Juvenile idiopathic arthritis, Biologic treatment, Safety therapy, Clinical practice

## Abstract

**Background:**

Biologic therapy has changed the prognosis of patients with juvenile idiopathic arthritis (JIA). The aim of this study was to examine the pattern of use, drug survival, and adverse events of biologics in patients with JIA during the period from diagnosis to adulthood.

**Methods:**

All patients included in BIOBADASER (Spanish Registry for Adverse Events of Biological Therapy in Rheumatic Diseases), a multicenter prospective registry, diagnosed with JIA between 2000 and 2015 were analyzed. Proportions, means, and SDs were used to describe the population. Incidence rates and 95% CIs were calculated to assess adverse events. Kaplan-Meier analysis was used to compare the drug survival rates.

**Results:**

A total of 469 patients (46.1% women) were included. Their mean age at diagnosis was 9.4 ± 5.3 years. Their mean age at biologic treatment initiation was 23.9 ± 13.9 years. The pattern of use of biologics during their pediatric years showed a linear increase from 24% in 2000 to 65% in 2014. Biologic withdrawal for disease remission was higher in patients who initiated use biologics prior to 16 years of age than in those who were older (25.7% vs 7.9%, *p* < 0.0001). Serious adverse events had a total incidence rate of 41.4 (35.2–48.7) of 1000 patient-years. Patients younger than 16 years old showed significantly increased infections (*p* < 0.001).

**Conclusions:**

Survival and suspension by remission of biologics were higher when these compounds were initiated in patients with JIA who had not yet reached 16 years of age. The incidence rate of serious adverse events in pediatric vs adult patients with JIA treated with biologics was similar; however, a significant increase of infection was observed in patients under 16 years old.

**Electronic supplementary material:**

The online version of this article (10.1186/s13075-018-1728-3) contains supplementary material, which is available to authorized users.

## Background

Juvenile idiopathic arthritis (JIA) comprises a group of diseases of unknown etiology that have in common arthritis in at least one joint persisting for at least 6 weeks in patients younger than 16 years of age [1]. JIA is the most frequent chronic rheumatic disease in childhood [2–4] and is classified into seven categories: systemic, persistent or extended oligoarthritis, rheumatoid Factor (RF) positive polyarthritis, RF-negative polyarthritis, enthesitis-related arthritis, psoriatic arthritis, and undifferentiated arthritis [1]. When uncontrolled, JIA leads to severe joint damage and impairment in skeletal maturation [5–7]. Fortunately, during the last decade, the arrival of biologics has dramatically changed the prognoses for these patients [8, 9]. A number of well-designed clinical trials, as well as cohort studies, have demonstrated that biologics are an effective option for patients with JIA who do not respond to or who cannot tolerate treatment with synthetic disease-modifying antirheumatic drugs (DMARDs) [10–12]. Some studies have shown that the sooner treatment is begun for JIA and the more aggressive it is, the better the outcomes obtained [13–16]. The ReACCh-Out cohort studied remission in patients with JIA in Canada and concluded that the probability of attaining remission with contemporary treatments within 5 years of diagnosis averaged about 50%, except for children with polyarthritis [17].

JIA is not confined to childhood, and 41% of patients require medication in their thirties, with some 28% maintaining high disease activity [18]. The transition period from pediatric- to adult-focused health care for adolescents with chronic conditions is attracting growing attention [19]. A recent study assessing the importance of transition to adult rheumatologic care in young people with JIA concluded that the maintenance of JIA diagnosis and DMARD therapy depended on the use of specialized care services [20]. Nowadays, young people with rheumatic diseases have a greater rate of survival, although high morbidity persists that could be avoided, in part, with multidisciplinary management [21]. Recently, several professional groups and international agencies have attempted to create consensus recommendations and guidelines trying to improve this situation [22, 23]. Data from the British Society for Rheumatology Biologics Register shows that tumor necrosis factor inhibitor (TNFi) therapies are an effective treatment option for adults with JIA, with a safety profile similar to that seen in rheumatoid arthritis (RA) [24]. However, because adults with JIA comprise a heterogeneous group of patients whose clinical evolution, need for, and response to treatments are not well studied, follow-up and monitoring of these patients are required [25]. The aim of the present study was to evaluate the pattern of use, the survival, and the safety of biologic agents in patients with JIA during the period from diagnosis to adulthood who are included in the BIOBADASER registry.

## Methods

### Study design

BIOBADASER (Spanish Registry for Adverse Events of Biological Therapy in Rheumatic Diseases) is a multicenter prospective observational study monitored annually, the main objective of which is long-term safety assessments of patients who undergoing biologic therapies [26, 27]. Established in February 2000, BIOBADASER is based on clinical practice. The quality of the database is ensured by a clear definition of its aim, an optimized number of variables, and an easy method of data collection that allows for consistency checks. Incompleteness and agreement of data with patient charts are assessed by online monitoring of the entire included population and by on-site annual audits of 10% of the patients registered. All detected errors and inconsistencies are corrected on the basis of results of said monitoring. The BIOBADASER registry is supported by the Spanish Medicines Agency, and the clinical studies were approved by the ethics committee of Hospital Clinic Barcelona. All patients included in the BIOBADASER registry, or their legal representatives, signed a written informed consent prior to inclusion.

### Population

For this analysis, we selected all adult patients included in the database diagnosed with JIA. Owing to the initial design of the registry (BIOBADASER was not initially designed as a specific registry for JIA), patients with JIA were classified into systemic/oligoarthritis/polyarticular JIA, JIA related to enthesitis, or psoriatic JIA. Patients were enrolled in the registry when biologic treatment was initiated, and they were followed prospectively and evaluated if an adverse event (AE) occurred or if a change in the biologic therapy was decided either owing to an AE or because of inefficacy. The analysis of this study includes data from 2000 to December 2015, and only those patients still under active follow-up at the end of the study period were included.

### Variables

The following data were collected: (1) patient data, including gender, date of birth, diagnosis, date of diagnosis, and comorbidities; (2) data on treatment, including types of biologics and dates of initiation and discontinuation, reason for discontinuation, concomitant antirheumatic treatment and tuberculosis (TB) prophylaxis; and (3) data on AEs, including date of occurrence, type and classification of AE according to the Medical Dictionary for Regulatory Activities [MedDRA]) v13 [28], severity, and outcome.

### Statistical analysis

The patients included were described using descriptive statistics indicated by the type and distribution of variables. Proportions, means, and SDs or IQRs were used to describe our population and the use of treatments. The incidence rate (IR) per 1000 patient-years with 95% CI was estimated by group. Results were expressed as IR with the 95% CI. Survival rates were defined as end of treatment for any reason. IR comparisons between age groups were obtained by Poisson regression. Log-rank tests were used to assess the equality of survivor functions across age groups, monotherapy vs combined therapy, and treatment groups. All analyses were performed using Stata version 13.1 software (StataCorp, College Station, TX, USA).

## Results

A total of 469 patients from the BIOBADASER registry, 46.1% of them women (*n* = 216), were classified as systemic/oligoarthritis/polyarticular JIA (70.5%), JIA related to enthesitis (25%), and psoriatic JIA (4.5%). The mean age was 34.5 years (SD = 15.3). The mean age at diagnosis was 9.4 years (SD = 5.3), and the mean evolution years was 24.1 (SD = 14.1). Uveitis was recorded in 12.6% of patients (50% of them antinuclear antibody [ANA]-positive). Overall, the ANA test result was positive in 30.2% of patients with systemic/oligoarthritis/polyarticular JIA, 14.3% of patients with psoriatic JIA, and 6.8% of patients with enthesitis. HLA-B27 was determined in 42.9% of patients, with 22% testing positive (59.8% in enthesitis-related arthritis, 23.8% in psoriatic arthritis, and 8.5% in systemic/oligoarthritis/polyarticular JIA). The mean age at the beginning of biologic treatment was 23.9 years (SD = 13.9), and the mean number of years that patients were treated with these compounds was 9.6 (SD = 3.8). Table 1 shows the baseline characteristics of patients included in this analysis.

**Table 1:** Baseline characteristics of patients included in the study

Variable	Median (IQR) (Years)	No. (%)
Total number of patients		469
Sex, female		216 (46.1)
Age^a^	32.8 [22.8–43.6]	
Age at diagnosis	10.3 [4.4–14.3]	
Age at the beginning of biological treatment, median [IQR]**	22.1 [13.9–32.5]	
Years of disease progression	22.5 [12.3–33.9]	
Time in treatment with biologic	9.7 [6.8–12.6]	
JIA categories:		
- Oligoarticular, polyarticular, and systemic		331(70.5)
- Enthesitis-related arthritis		117 (25)
- Psoriatic arthritis		21 (4.5)
Positive RF		32 (6.8)
ANA-positive		111 (23.7)
HLA-B27-positive:		
- Positive		103 (22)
- Negative		98 (20.9)
- Not done		268 (57.1)
Uveitis:		
- Without uveitis		410 (87.4)
- Uveitis, ANA-negative		30 (6.4)
- Uveitis, ANA-positive		29 (6.2)

*Abbreviations: ANA* Antinuclear antibodies, *HLA* Human leukocyte antigen, *JIA* Juvenile idiopathic arthritis, *RF* Rheumatoid factor

^a^Age at the end of BIOBADASER phase II (December 2015)

**Fig. 1:**
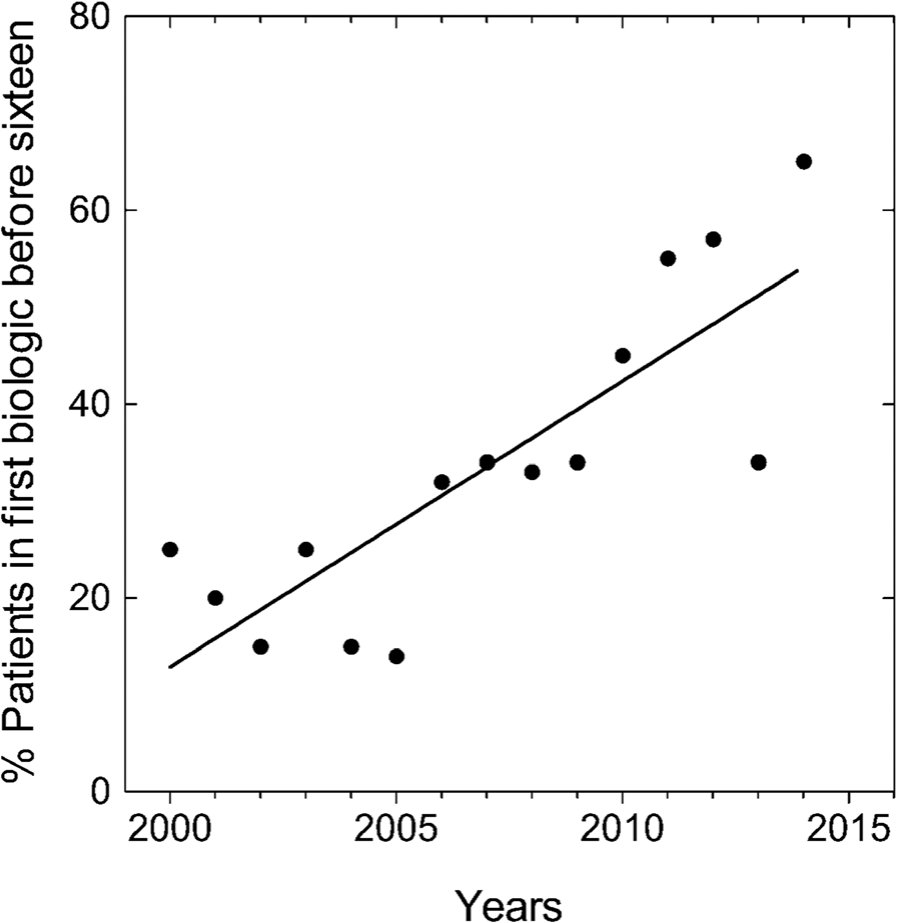
Dot plot showing the variations in the percentage of patients with juvenile idiopathic arthritis included in BIOBADASER annually who received their first biologic before age 16 since years 2000 to 2015

Table 2 shows the biologics and concomitant treatments. The most frequently used biologics as first-line treatments were etanercept (43.5%), infliximab (30.5%), and adalimumab (19%). Almost half of the patients included in the registry (42.4%) were receiving monotherapy with biologics, and corticoids were used in 32.3% of patients with JIA. With respect to the pattern of use, Fig. 1 shows the annual percentage of patients diagnosed of JIA from 2000 to 2015 whose treatment with biologics began at age 16 years or younger (*n* = 137). In 2000, 25% of patients received the first biologic at a pediatric age, with this percentage increasing linearly until reaching 65% in 2015. The mean age of patients who started biologic treatment before 16 years of age was 8.06 years (SD = 4.3).

**Table 2:** Biologics as first-line and subsequently, and concomitant therapy in patients with juvenile idiopathic arthritis

Drug	First-line	Second-line or later	Total
Etanercept	204 (43.5)	119 (25.8)	323 (34.7)
Infliximab	143 (30.5)	58 (12.6)	201 (21.6)
Adalimumab	89 (19.0)	108 (23.4)	197 (21.2)
Anakinra	8 (1.7)	15 (3.3)	23 (2.5)
Rituximab	0 (0.0)	72 (15.6)	72 (7.7)
Abatacept	5 (1.1)	28 (6.1)	33 (3.6)
Tocilizumab	16 (3.4)	40 (8.7)	56 (6.0)
Golimumab	2 (0.4)	13 (2.8)	15 (1.6)
Certolizumab	2 (0.4)	5 (1.1)	7 (0.8)
Canakinumab	0 (0.0)	2 (0.4)	2 (0.2)
Ustekinumab	0 (0.0)	1 (0.2)	1 (0.1)
Use of concomitant drugs			
- Monotherapy	177 (37.7)	217 (47.1)	394 (42.4)
- Methotrexate	225 (48.0)	184 (39.9)	409 (44.0)
- Glucocorticoids	155 (33.1)	145 (31.5)	300 (32.3)
- Leflunomide	29 (6.2)	26 (5.7)	55 (5.9)
- Sulfasalazine	26 (5.5)	13 (2.8)	39 (4.2)
- Gold salts	1 (0.2)	–	1 (0.1)
- Azathioprine	3 (0.6)	5 (1.1)	8 (0.9)
- Hydroxychloroquine	4 (0.9)	5 (1.1)	9 (1.0)

Data are expressed as number of patients (%)

Table 3 shows the type of biologic used in the initial treatment of patients younger vs older than 16 years old. Etanercept was the most frequently prescribed drug as a first-line treatment in patients under 16 years old (59.1%), whereas infliximab was the most often biologic used in those aged 16 years or older (40.4%). Maintaining this division, the reasons for suspension in both groups of ages were inefficiency (37.1% and 37.4%, respectively), followed by adverse effects (28.6% and 28%, respectively). The suspension rate due to disease remission was higher in patients who initiated biologic treatment before 16 years old (25.7%) than in those who began it at 16 years old or later (7.9%; *p* < 0.0001). A total of 266 (56.7%) patients received only one biologic, and 7.5% were treated with five or more such compounds.

**Table 3:** Characteristics of patients by age at the beginning of biologic treatment according to biologic compound, number of biologic drugs, and reasons for suspension

	< 16 Years	≥16 Years	*p* Value
Number of patients	137 (29.2)	332 (70.8)	
Age at the beginning of biological treatment	9.0 [4.2]	30.2 [11.4]	< 0.001
Years of disease progression	2.7 [3.1]	19.5 [2.1]	< 0.001
Biologic compound			
Etanercept	81 (59.1)	123 (37.1)	< 0.001
Adalimumab	31 (21.6)	58 (17.5)	
Infliximab	9 (6.6)	134 (40.4)	
Tocilizumab	8 (5.8)	8 (2.4)	
Abatacept	–	5 (1.5)	
Anakinra	6 (4.4)	2 (0.6)	
Certolizumab	2 (1.5)	–	
Golimumab	–	2 (0.6)	
Reason for suspension			
Inefficacy	26 (37.1)	80 (37.4)	< 0.001
Remission	18 (25.7)	17 (7.9)	
Adverse event	20 (28.6)	60 (28.0)	
Loss of tracking	4 (5.7)	20 (9.4)	
Pregnancy or gestational desire	–	13 (6.1)	
Other reasons	2 (2.9)	22 (10.3)	
Unknown	–	2 (0.9)	
Number of biologic drugs			
1 drug	83 (60.6)	183 (55.1)	0.244
> 1 drug	54 (39.4)	149 (44.9)	
2	27 (19.7)	67 (20.2)	
3	13 (9.5)	38 (11.5)	
4	11 (8.0)	12 (3.6)	
5 or more	3 (2.2)	32 (9.6)	

Data are expressed as mean [SD] or as number of patients (%). Chi-squared tests were used to compare distributions for categorical variables, and Student’s *t* tests were used for numerical variables

**Fig. 2:**
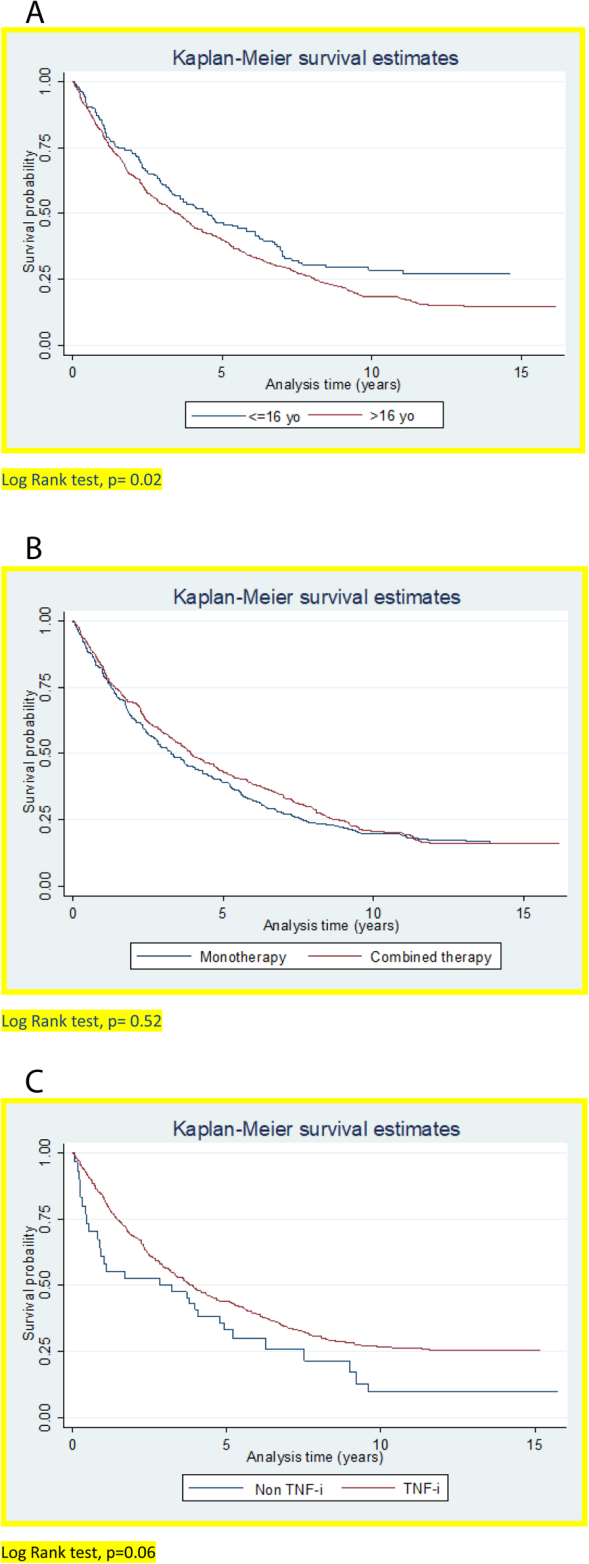
Kaplan-Meier survival curves. **a** Drug survival estimates by age (*p* = 0.02). **b** Drug survival estimates by monotherapy or combined therapy (*p* = 0.52). **c** Drug survival estimates by non-tumor necrosis factor inhibitor (non-TNFi) or TNF-i, only considering first and second lines of treatment (*p* = 0.06)

With respect to survival rates by age groups, retention of the first biologic was higher when it was started before 16 years of age (*p* = 0.02) (Fig. 2a). However, when survival rates were analyzed in patients who had received biologics as monotherapy or in combination, no significant differences were observed in terms of biologic retention (*p* = 0.52) (Fig. 2b). Regarding the therapeutic target, a nonsignificant tendency toward a better retention of TNFi compounds with respect to non-TNFi was observed (*p* = 0.06) (Fig. 2c) when they were analyzed as a first- or second-line treatment.

Table 4 shows that the most frequent AEs were infections, gastrointestinal disorders, skin and subcutaneous tissue disorders, and site-of-administration reactions. Regarding serious AEs, no differences were observed if the biologic therapy was initiated before age 16 years vs later. Conversely, the analysis of infections showed a significant increase in the IR in patients under 16 years of age: 253.2 (221.4–289.6) vs 136.0 (122.8–150.6) (*p* < 0.001). A total of 37.2% of infections occurred during the first year of treatment (only 4.0% in the first month). Additional file 1: Table S1 shows the frequency of infections classified by type in patients younger or older than 16 years old. Only one fatal event (mycoplasma pneumonia in a patient treated with anakinra) was recorded. The IRs of total AEs in the three categories in which BIOBADASER classified patients with JIA were as follows: 424.9 (399.8–451.7) for systemic/oligoarthritis/polyarticular JIA, 262.8 (232–297.9) in cases of enthesitis, and 252.9 (188.8–338.8) for psoriatic JIA (*p* < 0.001). Regarding serious AEs, the IRs were 44.1 (36.5–53.3), 36 (25.7–50.4), and 33.7 (15.2–75.1), respectively (*p* = 0.36). When patients with JIA were classified on the basis of biologic therapy with TNFi or non-TNFi, the IRs for total AEs were 345.0 (325.1–366.0) and 617 (541.7–702.8), respectively (*p* = 0.009). Regarding serious AEs, the IRs were 78.8 (54.8–113.4) and 37.1 (30.9–44.4), respectively (*p* = 0.924).

**Table 4:** Incidence of adverse events recorded in patients with juvenile idiopathic arthritis

	By age		*p* Value	Total
	< 16 years	≥16 years		
Total adverse events	457.6 (414.1–505.7)	346.9 (325.4–369.8)	< 0.001	373.2 (353.6–393.8)
Serious adverse events	35.7 (24.9–51.0)	43.2 (36.1–51.8)	0.347	41.4 (35.2–48.7)
Fatal adverse events	1.2 (0.2–8.4)	–	0.999	0.3 (0.0–2.0)
By system/organ class				
Infections and infestations	253.2 (221.4–289.6)	136.0 (122.8–150.6)	< 0.001	163.8 (151–177.6)
Gastrointestinal disorders	30.9 (21.0–45.4)	18.1 (13.7–24.0)	0.028	21.1 (16.9–26.5)
Skin and subcutaneous tissue disorders	17.8 (10.7–29.6)	21.1 (16.2–27.3)	0.797	20.3 (16.1–25.6)
General disorders and administration site conditions	21.4 (13.5–34.0)	20.0 (15.3–26.0)	0.566	20.3 (16.1–25.6)
Eye disorders	23.8 (15.3–36.8)	13.3 (9.6–18.4)	0.037	15.8 (12.1–20.5)
Musculoskeletal and connective tissue disorders	11.9 (6.4–22.1)	11.8 (8.4–16.7)	0.988	11.8 (8.7–16)
Surgical and medical procedures	3.6 (1.2–11.1)	13.7 (9.9–18.9)	0.025	11.3 (8.3–15.4)
Nervous system disorders	10.7 (5.6–20.6)	11.5 (8.1–16.3)	0.857	11.3 (8.3–15.4)
Blood and lymphatic system disorders	10.7 (5.6–20.6)	8.9 (5.9–13.2)	0.631	9.3 (6.6–13.1)
Renal and urinary disorders	3.6 (1.2–11.1)	11.1 (7.7–15.9)	0.061	9.3 (6.6–13.1)
Reproductive system and breast disorders	4.8 (1.8–12.7)	9.6 (6.5–14.1)	0.190	8.5 (5.9–12.1)
Injury, poisoning, and procedural complications	8.3 (4.0–17.5)	8.1 (5.4–12.3)	0.957	8.2 (5.7–11.8)
Respiratory, thoracic, and mediastinal disorders	11.9 (6.4–22.1)	5.9 (3.6–9.6)	0.083	7.3 (5–10.8)
Neoplasms: benign, malignant, and unspecified (including cysts and polyps)	3.6 (1.2–11.1)	5.9 (3.6–9.6)	0.422	5.4 (3.4–8.4)
Hepatobiliary disorders	2.4 (0.6–9.5)	5.9 (3.6–9.6)	0.225	5.1 (3.2–8.1)
Metabolism and nutrition disorders	1.2 (0.2–8.4)	5.9 (3.6–9.6)	0.120	4.8 (3–7.7)
Pregnancy, puerperium, and perinatal conditions	0.0	5.5 (3.3–9.2)	0.997	4.2 (2.5–7)
Vascular disorders	1.2 (0.2–8.4)	4.8 (2.8–8.3)	0.178	3.9 (2.3–6.7)
Psychiatric disorders	5.9 (2.5–14.3)	3.3 (1.7–6.4)	0.298	3.9 (2.3–6.7)
Ear and labyrinth disorders	2.4 (0.6–9.5)	3.0 (1.5–5.9)	0.783	2.8 (1.5–5.2)
Immune system disorders	2.4 (0.6–9.5)	2.6 (1.2–5.4)	0.916	2.5 (1.3–4.9)
Cardiac disorders	3.6 (1.2–11.1)	1.8 (0.8–4.4)	0.368	2.3 (1.1–4.5)
Endocrine disorders	2.4 (0.6–9.5)	1.5 (0.6–3.9)	0.583	1.7 (0.8–3.8)
Congenital, familial, and genetic disorders	0.0	1.8 (0.8–4.4)	0.403	1.4 (0.6–3.4)

Data represent the incidence (95% CI) × 1000 patients/yr

With respect to opportunistic infections, three cases of pulmonary TB and one case of disseminated TB were recorded; these patients had received chemoprophylaxis with isoniazid, except in one case owing to a negative Mantoux test result.

## Discussion

The most important findings of this work can be summarized as follows:

1. The use of biologics for the management of JIA during the pediatric years has consistently increased during the last 15 years.

2. In patients with JIA treated with biologics before 16 years of age, both the survival and drug withdrawal due to disease remission are higher than when these compounds are started during adulthood.

3. The safety of biologics in JIA is similar when used before or after 16 years of age in cases of serious AEs; however, in patients younger than 16 years old, infections are more frequent.

During the last 15 years, the advent of biologic drugs has changed the prognosis of and therapeutic approach to many rheumatic diseases, including JIA. As in adults, early initiation of biologic therapy in pediatric patients with JIA is important when control of the disease with conventional DMARDs is not achievable [29–31]. Patients with active JIA refractory to DMARDs and steroids are currently treated with TNFi or interleukin-6 antagonists or T-cell activation inhibitors in order to maintain inactive disease and the remission of JIA. Biologic drugs are typically well tolerated by children, and that—combined with early and aggressive therapy—yields optimal outcomes [9]. In this regard, in our present study, the pattern of biologic use showed that the annual percentage of patients with JIA who began treatment with these compounds before reaching 16 years of age increased almost threefold from 2000 to 2015. The biologics used in our registry of patients were, in order of frequency: etanercept, infliximab, and adalimumab. Infliximab was the second most used biologic in our series, with 30.5% of patients receiving this compound as the first biologic drug. Similarly, previous studies have reported the use of infliximab as a first biologic in patients with JIA ranged between 20.6% and 32% [24, 32]. When differentiating whether the treatment was started before or after 16 years old, it was found than 93% of patients (*n* = 134) initiated infliximab in adulthood. The reason for the significant use of infliximab, a TNFi without indication in any category of JIA, in patients with JIA in adulthood may be due to different factors. These might be that infliximab and etanercept were the first TNFi available, and for a period of time they were the only biologic available, to treat DMARD-resistant inflammatory arthritis, as well as the tendency of many rheumatologists to reclassify adult JIA as RA [20], where infliximab is indicated.

The variable course of JIA and the passage from adolescence to adulthood constitutes an important challenge for the physician. According to a study conducted by the American College of Rheumatology, 45% of pediatric rheumatologists are reluctant to treat patients aged 18 years and older, and 28% of adult rheumatologists are treating patients younger than 17 years old [33]. Various studies have reported that one-third of patients with JIA continue to present clinical disease activity into adulthood [34–36]. In addition, most patients with JIA who start biologic therapy during childhood reach adulthood with little evidence to support the benefits of continuing these treatments [37–39] or even of their long-term safety. This scenario implies a greater complexity in monitoring the safety of those drugs than is currently acknowledged. Studies based on routine clinical practice, as presented in this work, allow the assessment of treatment effectiveness and long-term adverse reactions in daily practice.

In our study, those patients who started treatment with biologics before the age 16 years presented with a percentage of drug suspension, owing to disease remission, greater than that in those who initiated these compounds at a later age (25.7% vs 7.9%, respectively). Different European cohorts have reported that male sex and earlier initiation of biologic therapy increased the likelihood of halting treatment owing to the onset of clinical remission [40]. With respect to drug survival, we found a significantly better retention rate when biologics were started before age 16 than in adulthood. The gap between diagnosis and biologic treatment initiation in the group of patients younger than 16 was 2.7 ± 3.1 years. A previous report [41] found no differences in the retention rates of the biologic therapy based on the age of treatment initiation in patients with JIA. In this study, the median age of patients who started biologic therapy was 16.2 ± 9.4 years old, with a gap between initiation of biologic treatment and diagnosis of 7.5 ± 4.9 years. This difference in start time of biologic treatment with respect to diagnosis might explain, at least in part, the divergent results in retention rates vis-à-vis age of biologic initiation between the two studies. In terms of drug survival, we found no differences between the use of biologics in monotherapy vs in combination, and when differentiated by age, our results were similar to those previously reported [42].

The aim of this study was to collect long-term outcome data on children receiving biologic agents for JIA, not only to assess drug survival but also to explore the reasons why biologic therapies had been discontinued. In our group of patients, inefficacy was the main reason for biologic therapy discontinuation regardless of age at drug onset, followed by AE and remission. In this regard, other series have found dissimilar results. Verazza et al. [43], in a series of 1038 patients with JIA treated with etanercept, found that the main cause of treatment discontinuation was disease remission, followed by inefficacy and AEs. Nevertheless, in a cohort of 301 patients with JIA, the most common reasons for stopping biologic treatment were AEs, with infusion reactions being the most frequently reported [44]. In a comparative study of adult and juvenile populations with inflammatory arthritis [42], the same biologic therapy profile as in our study was observed (infliximab vs etanercept) in both adults and children. Inefficacy was the most frequent reason for discontinuation of biologic therapy in both groups, being neuropsychiatric, gastrointestinal, and ocular complications, but not infections, as the most frequents AEs in the juvenile population. Although it may seem interesting to compare incidences of AEs and survival curves head-to-head for biologic treatments in adult patients, this would involve analyzing two different populations with clearly differentiated baseline pathologies and characteristics.

Regarding the limitations of our study, BIOBADASER was not specifically designed for JIA, and the categorization of these patients differs from the most currently used classification [1]. Because correct classification in categories is important in terms of therapeutic indications and prognosis, the new 2016 version of BIOBADASER classifies patients with JIA as systemic, persistent, or extended oligoarthritis; RF-positive polyarthritis; RF-negative polyarthritis; enthesitis-related arthritis; psoriatic arthritis; and undifferentiated arthritis [1]. Another limitation of our study is that we did not compare drugs individually. Nevertheless, our results included a safety comparison between TNFi and other biologic therapies. With respect to strengths of BIOBADASER, this registry allows the possibility of studying safety information in biologic treatments in a large cohort of patients with JIA followed in routine clinical practice by rheumatologists during a relevant period. BIOBADASER adds to the limitations of randomized clinical trials, which typically include a relatively low number of subjects followed for a short period of time, which hampers the ability of such studies to detect rare events and/or long-term side effects [45].

## Conclusions

In summary, thus far, there are few studies based on general clinical practice that focus on the safety of biologic treatments in patients with JIA. The prospective records of these adult patients with JIA treated with biologic therapy can contribute to improving knowledge about the behavior of this disease in adulthood. In our study, survival and suspension by remission of biologics were higher when these compounds were initiated in patients with JIA who had not yet reached 16 years of age. The IR of serious AE in child vs adult patients with JIA treated with biologics was similar.

## Additional file

Additional file 1: **Table S1.** Frequency of infections, classified by type and age. (DOCX 14 kb)

## Abbreviations

AE: Adverse event
ANA: Antinuclear antibody
BIOBADASER: Spanish Registry for Adverse Events of Biological Therapy in Rheumatic Diseases
DMARD: Disease-modifying antirheumatic drug
FER: Spanish Society of Rheumatology
HLA: Human leukocyte antigen
IR: Incidence rate
JIA: Juvenile idiopathic arthritis
RA: Rheumatoid arthritis
RF: Rheumatoid factor
TB: Tuberculosis
TNFi: Tumor necrosis factor inhibitor
