# Supplementary material for: Persistence and adverse events of biological treatment in adult patients with juvenile idiopathic arthritis: results from BIOBADASER
Source: Arthritis Res Ther. 2018 Oct 10;20:227. doi: 10.1186/s13075-018-1728-3 (PMC6235210; doi:10.1186/s13075-018-1728-3)
Supplement: Supplementary file 1 — Table S1. Frequency of infections, classified by type and age. (DOCX 14 kb) [file 13075_2018_1728_MOESM1_ESM.docx]

Additional file 1

Table S1 Frequency of infections classified by type and age.

|  | **< or = 16 years-old** | | **>16 years-old** | | **Total** | |
| --- | --- | --- | --- | --- | --- | --- |
| **Infections** | **N** | **%** | **N** | **%** | **N** | **%** |
| Upper respiratory tract infection | 42 | 19.72 | 63 | 17.12 | 105 | 18.07 |
| Urinary tract infection | 4 | 1.88 | 38 | 10.33 | 42 | 7.23 |
| Acute tonsillitis | 22 | 10.33 | 10 | 2.72 | 32 | 5.51 |
| Respiratory tract infection | 11 | 5.16 | 17 | 4.62 | 28 | 4.82 |
| Gastroenteritis | 10 | 4.69 | 16 | 4.35 | 26 | 4.48 |
| Pharyngitis | 15 | 7.04 | 9 | 2.45 | 24 | 4.13 |
| Herpes zoster | 5 | 2.35 | 18 | 4.89 | 23 | 3.96 |
| Herpes simple | 5 | 2.35 | 11 | 2.99 | 16 | 2.75 |
| Other | 99 | 46,5 | 186 | 50,5 | 285 | 48.89 |
| **Total** | 213 | 100 | 368 | 100 | 581 | 100 |
| Moment of infection | | | | | | |
| During the first month | 11 | 5.16 | 12 | 3.26 | 23 | 3.96 |
| During the first year | 86 | 40.38 | 107 | 29.08 | 193 | 33.22 |
| After the first year of treatment | 116 | 54.46 | 249 | 67.66 | 365 | 62.82 |
